# BETA-TESTING OF A CSMP APP: AN ASSESSMENT OF USABILITY, UTILITIES, AND CHALLENGES

**DOI:** 10.1093/geroni/igz038.2014

**Published:** 2019-11-08

**Authors:** Shinyi Wu, Kexin Yu, Mandong Liu, Haojun Jiang, Iris Chi

**Affiliations:** 1 University of Southern California, Los Angeles, California, United States; 2 Nanjing Agricultural University, Nanjing, Jiangsu, China (People’s Republic)

## Abstract

A beta-version of a CSMP app was designed to empower Chinese caregivers with the knowledge, skills, and self-efficacy needed for holistic health self-care at the convenience of using their mobile phone. The key app functions included interactive multi-media lessons and practice, scheduling of coaching sessions for problem solving, and extended readings. Four groups of 5 caregivers (totaling 20) sequentially participated in one-hour lab and one-week in-home testing of the app to identify usability, utilities and challenges of using the app. They also were invited to join a social media group chat to enable peer-to-peer communication for support and sharing. Using post-lab interviews and exit survey, the beta-testing allowed the research team to assess external user acceptance and feasibility of using the CSMP app in real-world settings, and helped the team explore the perceived training requirements, values, challenges, and improvement priorities to materialize learning CSMP curriculum in a mobile environment.

